# Polyamines: their significance for maintaining health and contributing to diseases

**DOI:** 10.1186/s12964-023-01373-0

**Published:** 2023-12-04

**Authors:** Mengjuan Xuan, Xinyu Gu, Juan Li, Di Huang, Chen Xue, Yuting He

**Affiliations:** 1 Department of Infectious Disease, The First Affiliated Hospital of Zhengzhou University, No. 1 Jianshe East Road, Erqi District, Zhengzhou, 450052 Henan China; 2 Department of Oncology, College of Clinical Medicine, The First Affiliated Hospital, Henan University of Science and Technology, Luoyang, 471000 Henan China; 3 Department of Child Health Care, The Third Affiliated Hospital of Zhengzhou University, Zhengzhou, 450000 Henan China; 4 Department of Hepatobiliary and Pancreatic Surgery, The First Affiliated Hospital of Zhengzhou University, Zhengzhou, 450052 Henan China

**Keywords:** Polyamine metabolism, Disease, Cancer, Oncogene, Mechanism

## Abstract

Polyamines are essential for the growth and proliferation of mammalian cells and are intimately involved in biological mechanisms such as DNA replication, RNA transcription, protein synthesis, and post-translational modification. These mechanisms regulate cellular proliferation, differentiation, programmed cell death, and the formation of tumors. Several studies have confirmed the positive effect of polyamines on the maintenance of health, while others have demonstrated that their activity may promote the occurrence and progression of diseases. This review examines a variety of topics, such as polyamine source and metabolism, including metabolism, transport, and the potential impact of polyamines on health and disease. In addition, a brief summary of the effects of oncogenes and signaling pathways on tumor polyamine metabolism is provided.

Video Abstract

**Supplementary Information:**

The online version contains supplementary material available at 10.1186/s12964-023-01373-0.

## Introduction

Polyamines, which are small polycationic compounds with a positive charge, are found in all types of living cells, including those of mammals, plants, and prokaryotes [1]. It is well known that there are three primary types of polyamines present in mammalian cells: putrescine (Put), spermidine (Spd), and spermine (Spm). The discovery of polyamines, specifically spermidine and spermine, dates back to 1678 when Antonie van Leeuwenhoek made the initial observation [2]. Throughout the twentieth century, their structure was characterized and their biosynthetic pathway was determined as a result of ongoing research [3]. Although all eukaryotic cells are capable of synthesizing the three major polyamines, polyamines can also be obtained through diet and other alternative sources [4], including intestinal bacteria. In addition, polyamines are essential for the growth and proliferation of mammalian cells and are intricately linked to biological processes, such as replication, transcription, translation, and post-translational modification, which modulate cellular proliferation, differentiation, apoptosis, and tumorigenesis [4, 5]. Briefly, polyamines within mammalian cells are irreplaceable, as the depletion of polyamines will completely halt cell growth and proliferation.

Numerous empirical studies support the claim that spermidine, the most abundant polyamine in mammals, acts as an elixir of life [6, 7]. Previous studies have demonstrated that polyamine concentrations tend to decrease with age, demonstrating a negative correlation with advancing age [8, 9]. One study, for instance, elucidated the relationship between polyamine levels and age, revealing that individuals aged 31–56 years and over 90 years have significantly lower total polyamine concentrations than those aged 60–80 years and older. Surprisingly, however, the concentrations of spermidine and spermine in the aforementioned two age groups are greater than in those aged 60 to 80 years, and individuals aged over 90 years have the highest relative proportion of spermine among these three age groups [10]. In this experiment, the relationship between polyamine concentration and age differed from the above statement. There were two primary factors that contributed to the observed results, namely, a small sample size of only 78 individuals, and the discrepant levels of polyamines in plasma as compared with those found in tissue [11]. Additionally, the administration of Spm has been demonstrated to markedly increase the lifespan of several model organisms (such as yeast, fruit flies, worms, and etc.), which suggests that spermidine may be a promising agent for enhancing health and longevity [8, 12].

It has been experimentally demonstrated that polyamines exert a crucial function in ensuring the survival of cells and promoting their growth [13, 14]. Consequently, the regulation of polyamine concentration and activity is of the utmost importance and is achieved via a complex network of pathways. The regulation of intracellular polyamine levels and activities is governed by pathways involved in polyamine synthesis, degradation, and transport, which have been extensively studied in bacteria, yeast, and higher organisms [15]. Consequently, fluctuations in polyamine concentrations and disruptions in polyamine metabolism will inevitably have negative effects on overall health, possibly even initiating the occurrence and progression of diseases, such as acute kidney injury, Alzheimer's disease, diabetes, etc. [16–18]. Furthermore, it is essential to acknowledge that numerous studies have elucidated notable alterations in the quantities and functions of polyamines in cancer [19, 20].

The primary objective of the present review is to examine the source and metabolic mechanism of polyamines in humans, investigate the functions and mechanisms of polyamines in both non-neoplastic and neoplastic diseases, and ultimately provide potential insights for the treatment of said ailments.

## The source and metabolism of polyamine

### Polyamine biosynthesis

**Fig. 1:**
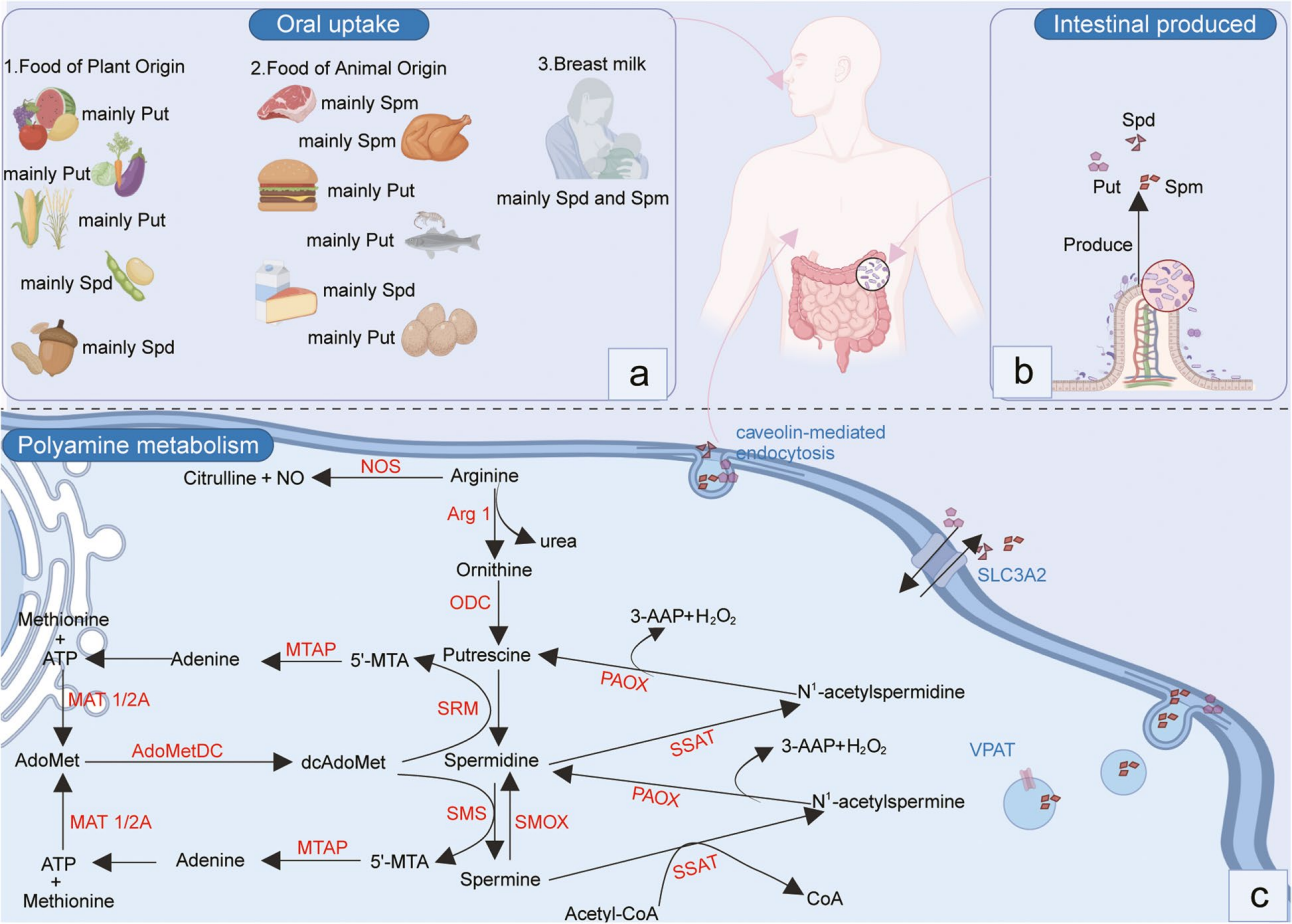
The source and metabolism of polyamine. **a** The presence of polyamines is ubiquitous in virtually all food sources. The main polyamines in breast milk are spermidine and spermidine, and the content and distribution of polyamines in plant-derived and animal-derived foods differ based on their respective categorizations. **b** The intestinal microbiota has the capability to produce polyamines. **c** The biosynthesis of polyamines commences with the conversion of L-ornithine into putrescine via the action of ornithine decarboxylase (ODC). Subsequently, the addition of an aminopropyl group, which is contributed by dcAdoMet, results in the formation of spermidine and spermine. In the process of polyamine catabolism, the enzyme SSAT are responsible for the acetylation of spermine and spermidine, resulting in the production of N1-acetylspermine and N1-acetylspermidine. SMOX can oxidize spermine directly to spermidine These metabolites are either secreted from cells or undergo reconversion back into spermidine and putrescine via the enzyme PAOX. Created with BioRender.com

The biosynthesis pathway of mammalian polyamine is depicted in Fig. 1 as a series of enzymatic reactions that lead to the production of these important intracellular components. Numerous microorganisms and higher plants have the ability to synthesize putrescine from agmatine via decarboxylation of arginine (ADC) [21, 22]. Despite the presence of evidence for ADC in mammals, Coleman et al. observed that ADC within mitochondrial extracts lack catalytic activity [23]. However, Wang et al. has indicated that the deficiency of ornithine decarboxylase (ODC) in ovine conceptus trophectoderm would ultimately result in an elevation of the activity of ADC [24]. Accordingly, the prevailing pathway for the de novo biosynthesis of putrescine in these species is via the enzyme ODC [25]. ODC is a pyridoxal phosphate-dependent enzyme and is frequently thought to be the rate-limiting factor in modulating the concentration of Put [26]. Ornithine, which is obtainable from the plasma and can also be generated intracellularly through arginase (Arg 1) activity, serves as the substrate for the synthesis of Put. Thus, it is possible that arginase, which is more widely distributed than other urea cycle enzymes, is present in extrahepatic tissues in order to ensure the availability of ornithine for polyamine synthesis. Arginase can, therefore, be regarded as the first step in the polyamine biosynthetic pathway [27].

A group of aminopropyl-transferases, specifically spermidine synthase (SRM) and spermine synthase (SMS), catalyzes the conversion of putrescine to spermidine and spermine during the synthesis of higher polyamines [28]. The aforementioned aminopropyl group originates from methionine and undergoes a two-step process consisting of conversion to S-adenosylmethionine and decarboxylation [27]. Subsequently, the decarboxylated S-adenosylmethionine (dcAdoMet) serves as an aminopropyl donor in a manner comparable to the use of S-adenosylmethionine as a methyl donor. Despite a close similarity in their responses, human SRM and SMS are discrete enzymes and exhibit stringent selectivity for their respective substrates [29]. Human SRM, a dimer comprised of two identical subunits, encompasses an active site within its C-terminal domain that shares a structural similarity with that of SMS [30]. Although the configurations of their active sites are comparable, they differ primarily in the constricted spatial allowance for the amine substrate within the framework of SRM, which guarantees putrescine is the only substrate for SRM [5].

During the synthesis of spermidine and spermine, the aminopropyl donor dcAdoMet produces the secondary metabolite 5'-methylthioadenosine (5'-MTA) [19]. In the methionine salvage pathway, MTA is initially phosphorylated by 5′-methylthioadenosine-phosphorylase (MTAP), after which it is converted into adenine (one of the primary components of ATP) and 5-methylthioribose-1-phosphate. Methionine metabolism involves the conversion of 5-methylthioribose-1-phosphate into a metabolite that can serve as a substrate for AdoMet synthesis through a cascade of enzymatic reactions. This is facilitated by methionine adenosyl-transferase 2 (MAT2), which catalyzes the final step of AdoMet synthesis by facilitating the ATP-dependent transfer of an adenosyl group to methionine. MTAP deficiency results in the inability of endogenous MTA to salvage methionine or adenine, resulting in impaired polyamine biosynthesis and the accumulation of dcAdoMet and 5'-MTA [31].

### Polyamine catabolism

It is generally accepted that the biochemical reactions catalyzed by spermidine synthase and spermine synthase are effectively irreversible. However, it has been established for many years that the transformation of spermine into spermidine and spermidine into putrescine can occur in vivo [28, 32, 33]. Spermine oxidase (SMOX) catalyzes the conversion of spermine to spermidine, 3-aminopropanaldehyde, and H_2_O_2_, with negligible effect on spermidine [33]. On the other hand, the enzyme acetylpolyamine oxidase (PAOX) catalyzes the transformation of N1-acetylspermidine into putrescine and N-acetyl-3-aminopropanaldehyde [34–37]. PAOX is also highly efficient at converting N1-acetylspermine to spermidine. The substrates required for PAOX-mediated reactions are produced through the catalytic activity of spermidine/spermine-N1-acetyltransferase (SSAT) [38]. The enzyme SSAT, encoded by the gene SAT1, typically exists at minimal concentrations but can be easily stimulated by increased levels of free polyamines [38]. SSAT facilitates the formation of N1-acetylspermine and N1-acetylspermidine, which may be extruded from the cellular environment or converted into 3-acetylaminopropanal, H_2_O_2_, and either Spd or Put, through the action of PAOX. The products generated via the reactions SMOX and PAOX, as well as acrolein, a toxic byproduct of spontaneous deamination of 3-aminopropanal [39] and Spm oxidation produced following cellular injury by copper-dependent oxidases present extracellularly [40], have been associated with renal insufficiency [41], ischemia–reperfusion [42] and injury cerebral infarction [43].

### Polyamine uptake

Aside from intracellular biosynthesis, the systemic availability of polyamines also depends on two other essential sources: extrinsic (oral) absorption via dietary intake and synthesis by intestinal microflora (Fig. 1) [44]. Surprisingly, it is believed that food consumption and endogenous production by intestinal microbiota are the primary sources of polyamines in healthy adult humans [45–47]. Following absorption by the gastrointestinal tract, polyamines are rapidly assimilated [48], resulting in a rapid elevation of portal vein concentrations and subsequent dispersion throughout the entire spectrum of organs and tissues [49]. Polyamines are ubiquitously present in all food types, such as shellfish with elevated polyamine concentrations per unit of caloric intake, albeit with substantial variation in their concentrations [50]. Given the abundance of polyamine sources, it appears that any effort to reduce the concentration of polyamines in order to promote health or alleviate disease must involve the elimination of all polyamine sources. Otherwise, the residual sources of polyamines could compensate for the maintenance of optimal polyamine levels [45].

### Polyamine transport

Due to the protonation of the primary and secondary amino groups of polyamines at the physiological pH of the extracellular environment [51], polyamine is conceptually incapable of diffusing passively through cellular membranes. While there exists a certain degree of comprehension regarding the polyamine transport, further researches are required to substantiate the precise transport mechanism and the transportation carriers involved. There have been three postulated models regarding the transport of polyamine, which include glypican-mediated endocytosis, plasma transport and vesicular sequestration, and caveolin-mediated endocytosis [52]. The glypican-1 molecule possesses a side chain consisting of heparan sulfate that can bind with Spm and facilitate its transport into the cytoplasm [53]. It has been hypothesized that polyamine transport may also involve transporters on the plasma membrane, which are similar to those found in yeast and bacteria [54]. Solute carrier (SLC) transporters and ATP transporters (such as ATP13A2 and ATP13A3) serve as a mediator for the transport of polyamine in mammalian cells [52, 55–57]. For instance, SLC family 3 member 2 (SLC3A2) facilitates the export of acetylated polyamines through a reaction involving the exchange of polyamines with arginine [58]. In addition, the finding of a vesicular polyamine transporter (VPAT, also known as SLC18B1) in astrocytes [59] and mast cells [60] highlights the potential neuro- and immune-modulatory effects of this transport mechanism [60, 61]. Moreover, the activated K-RAS modulates polyamine transport in colon cancer cells by regulating the expression of caveolin-1, which exerts a suppressive effect on caveolar endocytosis [62]. Therefore, the accurate delineation of the polyamine transport system will generate novel perspectives regarding the management and prognosis of certain diseases.

## The effect and mechanism of polyamine on disease

Growing evidence suggests that polyamines exert a substantial effect on the pathogenesis and progression of numerous diseases. In this regard, it has also been established that polyamines have diagnostic value in certain conditions, including cardiovascular disease [63, 64] and metabolic syndromes [65, 66]. Furthermore, their prognostic potential in predicting disease outcomes has also been demonstrated. Tables 1 and 2 provide extensive data on the properties and potential biological functions of polyamines in both neoplastic and non-neoplastic diseases.

**Table 1:** The regulation and function of polyamine in carcinogenesis

Neoplasm type	Target gene	Regulation	Expression	Associated protein	Inhibitor	Concentration/Activity	Polyamine level	Function	Refs
OC	MYC	positive	High	ODC	DFMO	↓	↓	inducing apoptosis through regulation of AP-1 by DFMO	[67]
	/	/	/	ODC	DEMO + aza	↓	↓	Increasing anti-tumor M1 macrophages through DFMO and aza	[68]
	/	/	/	ODC	DFMO	↓	↓	enhancing the cytotoxicity of PARP inhibitors via DFMO	[69]
	/	/	/	ODC	SBP-101	↓	↓	suppressing OC via SBP-101	[70]
HCC	MAT1A	positive	Low	MATI/III	/	↓	↓	increasing HCC cell proliferation	[71–73]
	MAT2A	positive	High	MATII	/	↑	↑	activating MAT2A in turn	[74]
	/	/	/	GSK-3β	SSAT	↓	↓	suppressing tumor progression and metastasis	[75]
	/	/	/	HDAC4	Spd	↓	↑	enhancing autophagy flux	[76]
GC	SMO	positive	High	SMO	/	↑	↓	promotes H. pylori-induced carcinogenesis	[77]
	/	/	/	SMO	/	↑	↓	increasing gastric cancer risk by H. pylori-induced overexpression of SMO	[78]
	CagA	positive	High	SMO	/	↑	↓	increasing malignant transformation through CagA	[79]
	AMD1	positive	High	AMD1	SAM486A	↓	↓	suppressing tumor growth by inhibiting AMD1	[80]
CRC	MAT2A	positive	High	MATII	/	↑	↑	activating MAT2A in turn	[74]
	MYC	positive	High	ODC	/	↑	↑	activating ODC through mutant APC	[81–83]
	APC	positive	High	ODC	DFMO	↓	↓	suppressing colorectal carcinogenesis by DFMO	[83]
	/	/	/	GSK-3β	SSAT	↓	↓	suppressing tumor progression and metastasis	[75]
	K-RAS	negative	High	Caveolin-1	/	↓	↑	boosting polyamine uptake via caveolin-1 phosphorylation	[84]
PDAC	/	/	/	EP300	Spd	↓	↑	inhibiting EP300 via Spd	[85–87]
	MTAP	negative	Low	ODC	/	↑	↑	increasing ODC through the loss of MTAP	[88, 89]
PCa	/	/	/	MTAP; SSAT	MTDIA + BENSpm	↓;↑	↓	Delaying or preventing PCa recurrence	[90]
	PTEN	negative	Low	AdoMetDC	/	↑	↑	promoting tumor growth	[91]
	MYC	positive	Low	ODC	PGC1-α	↓	↓	suppressing PCa	[92]
Neuroblastoma	MYCN	positive	High	ODC	DFMO	↓	↑	inhibiting MYCN-induced Neuroblastoma initiation and progression by DFMO	[93–96]
	MYCN	positive	High	SLC3A2	/	↑	↑	increasing polyamine synthesis by regulating SLC3A2	[97]
	MYC	positive	High	ODC	DFMO + AMXT-1501	↓	↓	emphasizing the necessity of polyamines in Neuroblastoma	[98]
	MYC	positive	Low	ODC	/	↓	↓	inhibiting polyamine metabolism by glucose deprivation	[99]

**Table 2:** The regulation and Function of polyamine in non-neoplastic diseases

Non-neoplastic Diseases	Organism	Tissue	Associated protein/cytokine	Function	Treatment	Concentration/Activity	Effect	Ref(s)
Circulatory system	mice	vessel in gastric cancer	ODC	biosynthesis of polyamine	DFMO	↓	decreasing the vessel density	[100]
	mice	artery	ODC	biosynthesis of polyamine	extracellular spermidine	↓	increasing NO bioavailability	[101, 102]
	mice	heart	TNF-α	inflammation of cardiomyocytes	spermidine	↓	decreasing the passive stiffness of cardiomyocytes	[63, 103]
	rats	heart	AMPK	activation of autophagy	/	↑	promoting autophagy by Spd-activated AMPK	[104]
	rats	heart	SIRT1	biogenesis of mitochondrion	/	↑	improving mitochondrial biogenesis by Spd	[105]
Alzheimer’s disease	mice	cerebrum	Arg 1; ODC	biosynthesis of polyamine	/	↑	inducing memory loss	[106, 107]
Asthma	mice	lung	SSAT; SMO	catabolism of polyamine	MDL72.527	↓	airway epithelial injury	[108]
	mice	lung	ODC	biosynthesis of polyamine	/	↑	airway hyperresponsiveness	[109]
Obesity	mice	WAT	SSAT	catabolism of polyamine	/	↑	increasing lipid oxidation	[110–112]
	drosophila	abdomen	SPDSY; SPMSY	biosynthesis of polyamine	/	/	regulating triglyceride storage	[113]
	mice	WAT	SSAT	catabolism of polyamine	/	↑	decreasing fatty acid synthesis	[114]
Pancreatitis	rats	pancreas	SSAT	catabolism of polyamine	/	↑	increasing trypsinogen activation	[115–117]
Psoriasis	mice	skin	Arg 1	biosynthesis of polyamine	nor-NOHA	↓	inhibiting self-RNA sensation within keratinocytes	[118]

### Non-neoplastic diseases

#### Polyamines in cardiovascular protection

Polyamines are essential to the angiogenesis process, which occurs in response to tissue damage or tumor growth [4]. It has been demonstrated that the inhibition of polyamine synthesis inhibits angiogenesis in both gastric ulceration models [119] and tumor models [100, 120].

**Fig. 2:**
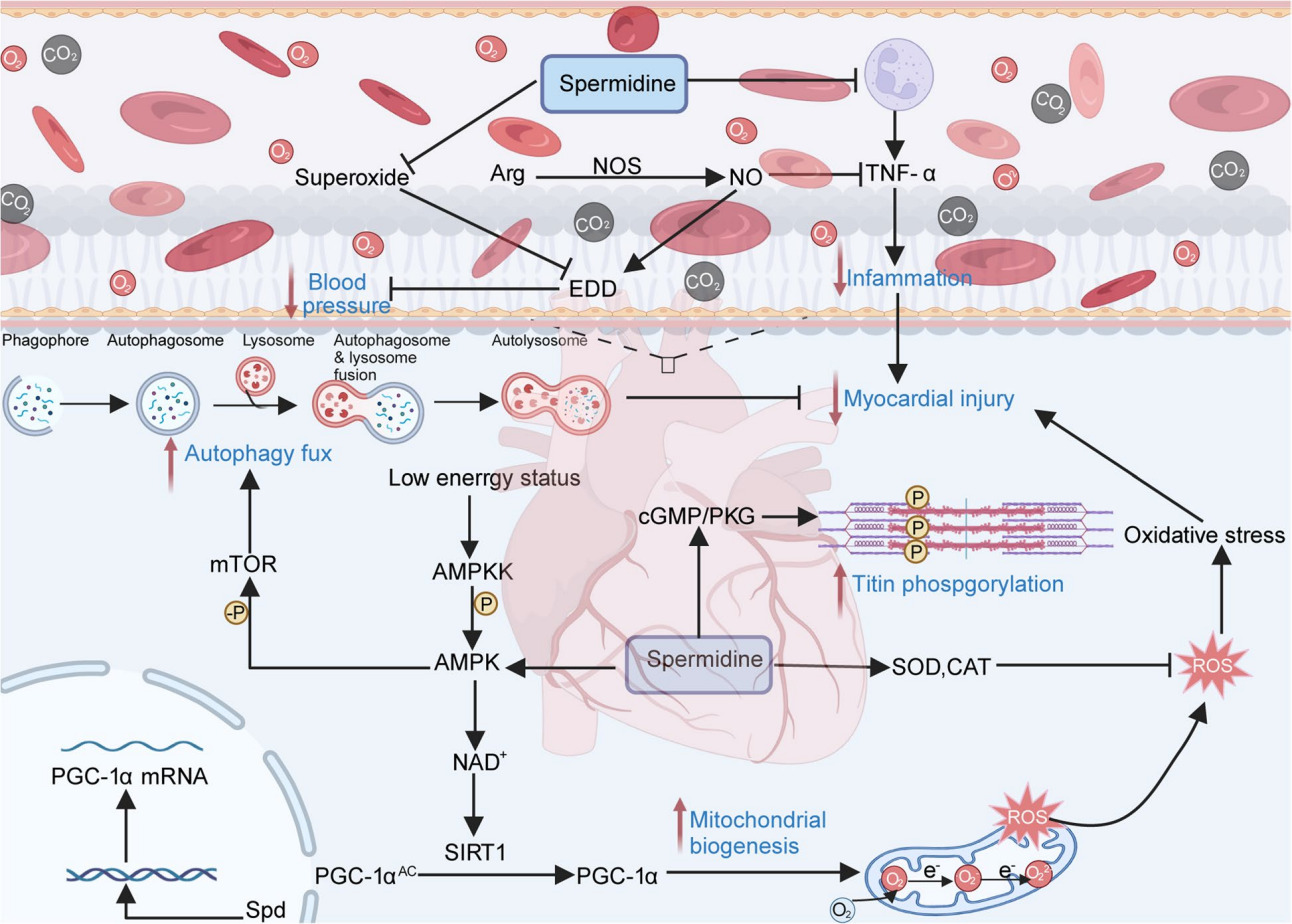
Cardiovascular protective effect of polyamines. (I) Spd effectively reinstates the NO-mediated endothelium-dependent dilation (EDD) by augmenting the level of NO bioavailability and abating oxidative stress, therefore reducing blood pressure. (II). Spd increases autophagy flux through the AMPK-mTOR signaling pathway, contributing to the stimulation of cardiomyocyte protective autophagy. (III). The structure and function of cardiomyocytes can be improved through Titin phosphorylation and mitochondria formation. And Spd can activate mitochondrial biogenesis through SIRT1-mediated PGC-1α deacetylation. (IV). Spd effectively suppresses the release of TNF-α by immune cells, consequently lowering levels of subclinical chronic inflammation and ultimately preventing the onset of myocardial injury. Created with BioRender.com

The process of aging induces two critical alterations in arteries, which markedly elevate the susceptibility to cardiovascular diseases (CVD): 1. The rigidity of the major elastic arteries (viz*.* the aorta and carotid arteries) and 2. The emergence of vascular endothelial dysfunction [121, 122]. Arterial stiffening results from age-related changes in the arterial wall, including increased collagen deposition, decreased elastin content, and cross-linking of these and other structural proteins via the formation of advanced glycation end products (AGEs) [123]. Age-related vascular endothelial dysfunction is primarily the result of decreased nitric oxide (NO) bioavailability, as indicated by impaired endothelium-dependent dilation (EDD) mediated by NO [124, 125]. LaRocca et al., suggested that spermidine has a remarkable anti-aging effect on arteries, which is due to its ability to increase the bioavailability of nitric oxide, reduce oxidative stress, alter structural factors, and enhance autophagy [101]. In addition, Eisenberg et al., concurred that spermidine administration promoted myocardial autophagy, mitophagy, and mitochondrial respiration, while simultaneously enhancing the mechanical and elastic properties of cardiomyocytes in vivo, which was accompanied by elevated titin phosphorylation and the suppression of subclinical inflammation [63]. Following the experiments conducted by Eisenberg et al., numerous researchers have endeavored to determine the precise mechanism by which spermidine promotes cardiac protection. As evident from the studies conducted by Yan et al., increasing autophagy via Spd-mediated targeting of the AMPK/mTOR signaling pathway improves cardiac dysfunction after myocardial infarction [104]. Notably, the aforementioned conclusions were all derived from in vivo experiments conducted on mice. Holbert et al. definitively highlighted that bovine serum amine oxidase in vitro experimentation can catalyze the oxidation of exogenous polyamines and subsequently trigger the release of reactive oxygen species (ROS), leading to the induction of autophagy [126]. Therefore, it is challenging to establish the particular mechanism of polyamines, particularly Spd, in facilitating cardiac protection. In 2020, Wang et al., conducted a study proving that Spd stimulates mitochondrial biogenesis [105]. The protective effect of Spd on the cardiovascular system has been confirmed in quite a few studies [102, 103]. This activation occurred as a result of SIRT1-mediated deacetylation of PGC-1α, which subsequently led to the alleviation of cardiac aging. Multiple other studies have verified the cardioprotective properties of spermidine on the cardiovascular system [127–129]. Therefore, spermidine is a potentially effective nutraceutical intervention for the mitigation of arterial aging and the prevention of age-related cardiovascular diseases (Fig. 2).

#### Polyamines in alzheimer's disease

Alzheimer's disease (AD) is recognized as the leading cause of cognitive dysfunction or dementia in individuals aged 65 and older and is rapidly emerging as one of the most expensive, lethal, and burdensome diseases of the twenty-first century [130, 131]. Alzheimer's disease is characterized by the deposition of beta-amyloid (Aβ) plaques and subsequent memory loss [132, 133]. The initial neuronal impairment in Alzheimer's disease patients typically occurs in regions of the brain responsible for cognitive processes such as memory, language, and thought. This, in turn, results in the emergence of early symptoms that typically involve difficulties in these particular cognitive domains [134, 135].

Considering the close association between the genes expressed by glial cells and AD, genetic studies suggest that AD research should shift its focus from neurons to glial cells and neuroinflammation [136]. Evidence from prior studies suggests that Put has the ability to produce GABA via the enzyme MAO-B (monoamine oxidase B) in astrocytes, which may pose a risk for memory impairment [137, 138]. In addition, a recent study demonstrates that the administration of β-amyloid induces a noteworthy upregulation of Put and GABA expression within astrocytes through the activation of specific intracellular signaling cascades [137]. Arg 1 and ODC are enzymes that catalyze the putrescine synthesis process, and inhibiting their activity would lead to a significant reduction in the production of putrescine. Therefore, inhibition of specific enzymes represents a potential therapeutic avenue for ameliorating cognitive impairments and potentially reducing β-amyloid plaque burdens [106, 107].

#### Polyamines in asthma

Asthma is a respiratory disorder characterized by intermittent bronchospasm, which results in dyspnea and wheezing [139, 140]. It is characterized by airway inflammation, airway hyperresponsiveness, and mucus hypersecretion, all of which contribute to variable airflow obstruction. The heterogeneous underlying inflammatory mechanisms in asthma further add complexity to the disease [140]. In this regard, elevated polyamine concentrations have been observed in the circulation of individuals experiencing asthma attacks, in addition to significantly elevated polyamine concentrations in bronchoalveolar lavage fluid collected from asthmatic patients [109, 141, 142].

The pathologic characteristics of asthma are intimately connected to the pathologic reactions and morphologic alterations of inflamed and structural cells. Moreover, the interaction between polyamines and immune and structural cells in the context of asthmatic pathophysiology implicates the polyamine pathway as having a beneficial effect on asthmatic responses [143]. SSAT and SMOX play significant roles in the process of polyamine catabolism [33, 38]. Knocking-down of the genes of these enzymes results in a decrease in polyamine catabolism, ultimately leading to an increase in the concentration of spermidine and spermine which will result in the induction of stress and apoptosis in bronchial epithelial cells [108]. In addition, research has shown that upregulation of Arg1 expression results in an increase in polyamine synthesis, ultimately leading to the development of airway hyperresponsiveness, which may be partially attributed to the inhibiting effect of Arg1 overexpression on NO synthesis [109]. Inflammatory cells can release a number of inflammatory factors that result in trachea spasms and severe asthma attacks [144]. Furthermore, it has been discovered that polyamines enhance the disease-causing capacity of cells involved in inflammatory responses, specifically mast cells and granulocytes such as eosinophils and neutrophils, by stimulating the liberation of their pro-inflammatory agents or extending their lifespan [143, 145, 146]. The underlying mechanisms by which polyamine contributes to the onset and progression of asthma have the potential to facilitate the development of therapeutic interventions.

#### Polyamines in obesity

Obesity constitutes a hazard in relation to a multitude of ailments encompassing metabolic morbidities (such as type 2 diabetes and hepatic steatosis), cardiovascular infirmities (such as coronary heart disease and cerebrovascular accidents), neoplastic disorders (such as hepatocellular and renal cell carcinoma), depressive illnesses, and other debilitating conditions, all of which have a negative effect on the health and lifespan of individuals [147–149]. The majority of patients may not benefit from traditional and invasive therapeutic approaches [150]. Polyamine exerts an extensive role in energy metabolism, particularly in regulating lipid metabolism, consequently representing a pivotal area of focus in the exploration of obesity and its associated ailments.Numerous studies have investigated the relationship between polyamines and energy metabolism, as well as the effects of variations in the enzymes involved in polyamine metabolism on body mass. The mechanism by which Spm exerts its anti-obesity effects was elucidated in the present study by Nakatani et al. [151]. In particular, Spm promotes β-oxidation [110–112] and suppresses pre-adipocyte differentiation, thereby reducing lipid accumulation [151]. The article by Sadasivan et al., demonstrated the significant efficacy of intraperitoneal administration of Spm in mice fed a high-fat diet, as evidenced by a 24% decrease in body weight and a 57% decrease in white adipose tissue (WAT) in comparison to the untreated control group [112]. A significant negative association between spermidine consumption and obesity was demonstrated by the findings of an epidemiological study [152]. Additionally, it was discovered that the administration of spermidine may lead to varying degrees of improvement in the weight status of mice whose obesity was induced by a high-fat diet [153, 154]. In addition, enzymes involved in the metabolism of polyamines regulate the storage site of triglycerides [113] and inhibit fatty acid synthesis [114]. The discoveries presented herein offer fresh perspectives on the pharmacodynamics of polyamine, and its suitability as a curative modality for obesity management.

#### Polyamines in pancreatitis

Acute pancreatitis is a prevalent inflammatory disorder of the exocrine gland, the pancreas, which manifests as severe abdominal pain and multi-organ dysfunction [155]. It can precipitate pancreatic tissue death [156] and persistent systemic dysfunction, resulting in a 1–5% mortality rate [155]. Due to the severe complications and increased mortality associated with pancreatitis, as well as the fact that spermidine is found in the highest concentration in pancreatic tissue [157] and polyamines are involved in the processes of cell proliferation and apoptosis [13, 14], it seems plausible that the deficiency of polyamines in individuals may serve as a catalyst for the onset and progression of pancreatitis.

It is essential to emphasize that SSAT is a key enzyme in polyamine catabolism, facilitating the acetylation of spermidine and spermine by transferring acetyl groups from CoA to produce acetylpolyamines [38]. In the previous two experiments, zinc administration was used to induce pancreatitis in transgenic rats genetically modified to overexpress the SSAT gene under the control of the inducible mouse metallothionein I promoter [115, 116]. Both of the aforementioned studies came to the conclusion that the catabolism of polyamines caused pancreatitis. The disagreement between the two groups of researchers relates to their divergent perspectives regarding the precise mechanism of pancreatitis. The former group hypothesized that polyamines function as protease inhibitors and demonstrated that SSAT activated trypsinogen while treatment with polyamine analogs prevented trypsinogen activation [115]. In contrast, the latter group hypothesized that H_2_O_2_ (a metabolite of acetylated polyamine) caused pancreatitis, but they disproved this theory by administering PAOX inhibitors, which claimed the inflammatory process was unrelated to the production of H_2_O_2_ [116]. The administration of polyamines has been shown to prevent pancreatitis, and the supplementation of polyamine analogs has also been observed to alleviate complications associated with severe pancreatitis [115–117, 158].

#### Polyamines on psoriasis

Psoriasis, a persistent and chronic inflammatory condition, has been discovered to exhibit a state of heightened arginine metabolism and exaggerated production of polyamines [118]. In particular, the absence of protein phosphatase 6 (PP6) hinders the maintenance of skin homeostasis, and this phenomenon has been shown to be associated with increased transcription of ARG1 via phosphorylation and activation of the C/EBP-β transcription factor, as well as an increased production of polyamines [118]. It is believed that the addition of cationic polyamines, which have a propensity for binding with nucleic acids, accelerates the internalization of self-RNA by psoriatic keratinocytes and the recognition of self-RNA by myeloid dendritic cells [118]. Significantly, the improvement of this inflammatory condition has been demonstrated through the restoration of the urea cycle with the nor-NOHA arginase inhibitor or administering DFMO as a treatment, offering a novel therapeutic approach for psoriasis [17].

### Neoplastic diseases

#### Ovarian cancer

According to ovarian cancer statistics in the United States in 2022, ovarian carcinoma (OC) comprises the leading cause of death among reproductive-related malignancies, with a prevalence of ~ 20,000 documented cases and ~ 13,000 fatalities per year [159]. More than 70% of OC patients are diagnosed at advanced stages (III–IV), placing ovarian cancer as the fifth leading cause of cancer-related deaths among women in developed nations [160]. Thus, the involvement of polyamines in the timely detection and subsequent management of ovarian cancer demonstrates great promise and potential.

Elevated levels of putrescine, spermidine, and spermine have been found in the urine of individuals with OC [161, 162]. Correspondingly, they have been utilized as biomarkers for the early detection of ovarian cancer [163, 164]. In addition, Fahrmann et al., presented findings of a distinct pattern of polyamines identified in blood samples, which holds significant potential for the early detection of OC [165]. In addition, this polyamine signature serves as a valuable complement to CA125 (human mucin 16, known as the most optimal marker for the early detection of OC) in identifying a greater number of ovarian cancer cases that would have been missed by CA125 alone [165, 166]. Given the propensity of polyamine to promote the proliferation and invasion of tumors, reducing its activity and concentration would be a crucial therapeutic intervention. As an inhibitor of the rate-limiting enzyme ODC in polyamine biosynthesis, DFMO (also known as α-difluoromethylornithine) is reported to induce apoptosis by regulating AP-1 signaling through JNK phosphorylation [67]. In addition, DFMO has demonstrated the ability to enhance the anticancer potential of co-administered pharmaceutical agents, such as 5-azacytidine [68] and poly (ADP-ribose) polymerase (PARP) inhibitor [69]. The co-administration of DFMO and 5-azacytidine upregulates M1 macrophage, resulting in a marked improvement in the survival rate of the combined therapeutic modality. In addition, the concomitant utilization of DFMO and inhibitors of PARP confers not only enhanced susceptibility of cancer cells to PARP inhibitors, but also increased cytotoxicity of the cisplatin. Moreover, the polyamine analog SBP-101(diethyl dihydroxyhomospermine) inhibits tumor cells by decreasing the activity of ODC [70]. In a word, the comprehensive investigation into the level and metabolism of polyamine will bring about new insights in diagnosis and treatment of OC.

#### Hepatocellular carcinoma

Primary hepatic carcinoid tumor, which predominantly results from hepatocellular carcinoma (HCC), imposes a severe economic and health burden on society, especially in China and other Asian countries/regions due to the high prevalence of chronic hepatitis B virus (HBV) infection [167]. HCC is a highly prevalent malignancy that ranks as the fourth leading cause of cancer-related mortality and the second leading cause of years of life lost due to cancer worldwide [168–170]. However, there is a lack of consensus regarding the function and mechanism of polyamines within the hepatic tissue and adjacent normal tissues of HCC-afflicted individuals. Thus, further investigation is necessary to delve more comprehensively in greater depth.

Polyamines exert regulatory control over the progression of the cell cycle and inhibit polyamine biosynthesis, resulting in cell cycle arrest and apoptosis [171], as previously described. A sudden increase in polyamine biosynthesis could potentially promote the rapid proliferation of preneoplastic and neoplastic hepatocytes [71–73]. During the progression of hepatocarcinogenesis in rats, there is a gradual increase in the expression of the ODC gene, as well as a concomitant elevation in ODC activity and polyamine synthesis [172, 173]. A similar upregulation of genes involved in polyamine synthesis is also observed in human hepatocellular carcinoma [174]. Frau et al., supported that SAM, acting as the principal supplier of methyl groups and a precursor for the synthesis of polyamines, interferes with polyamine synthesis via inhibition of ODC activity, which is partially dependent on the accumulation of 5'-MTA [71]. However, Lauda et al., demonstrated that increased expression of MAT2A, which encodes for the enzymatic component of MAT that catalyzes the formation of SAM [175], leads to more SAM for polyamine biosynthesis. The elevation in polyamine, specifically putrescine in this instance, resulted in a upregulation of mRNA expression levels for MAT2A and ODC, consequently augmenting the proliferative potential of the neoplastic cell [74]. Strikingly, in mice exposed to a potent chemical carcinogen, the administration of spermidine has been found to inhibit the development of HCC [76]. The selective aggregation of truncated microtubule-associated protein 1S (MAP1S) in response to mitotic arrest results in the disruption of mitochondrial function on the mitotic spindle, ultimately leading in mitotic cell death [176]. Accordingly, MAP1S has been found to interact with HDAC4 via a designated domain, and inhibiting HDAC4 results in increased acetylation and enhanced stability of MAP1S [177]. Spermidine enhances the acetylation and stability of MAP1S while also inducing autophagy flux via the depletion of cytosolic HDAC4, thereby reducing the association between MAP1S and HDAC4 [76]. Thus, the characterization of the autophagic flux induced by MAPS1 in mitigating liver cancer in murine models is likely to provide a novel strategy for harnessing MAP1S-triggered autophagic flux for the prevention, delay, or treatment of HCC [76]. In addition, Wang et al. discovered that the β-catenin pathway, which facilitated HCC cells growth and metastasis, can be inhibited through the upregulation of SSAT. This upregulation was strongly associated with a decrease in polyamines, leading to the inhibition of β-catenin translocation into the nucleus [75].

#### Gastric cancer

According to current statistics, gastric cancer (GC) stands as the fifth most prevalent form of cancer worldwide and the fourth primary mortality-inducing neoplasm [178, 179]. Significant regional disparities exist in the prevalence of GC, with notable occurrences in East Asian countries such as Japan and Northern Europe [179]. In addition, there is growing concern regarding the escalating incidence of GC among those under 50 years old [180]. Prior studies have established a link between polyamine metabolism and the infection of gastric epithelial cells, as well as the increased risk of GC mediated by *Helicobacter pylori* (Hp).

Spermine Oxidase (SMOX) is a crucial enzyme in the metabolism of polyamines in the gastrointestinal tract due to its efficient regulation of spermidine and spermine concentration, which is necessary for maintaining proper physiological function [181]. Numerous studies have elucidated that SMOX can trigger the occurrence of GC by instigating DNA damage [78, 182, 183]. The precise mechanism underlying DNA damage caused by SMOX in Hp-associated gastric cancer requires further elucidation in scholarly literature. The activation of the β-catenin oncogenic signaling pathway in gastric epithelial cells stimulated by Hp is facilitated by the mediator SMOX, as reported by Sierra et al. [77]. As indicated in 2011, there is also the opinion that the pathogenesis of Hp-induced gastric carcinogenesis is associated with the oncoprotein cytotoxin-associated gene A (CagA) of microbiological origin [79]. AMD1, also known as S-adenosylmethionine decarboxylase (AdoMetDC), is one of the key enzymes of polyamine biosynthesis that has a tumorigenic effect on human gastric cancer and affects patient prognosis [80]. In conclusion, it is evident that polyamines play a complex and sophisticated regulatory role in the occurrence and mechanism of Hp-induced GC. The investigation of the regulatory pathway of polyamines in the pathogenesis of gastric cancer and the search for novel polyamine inhibitors have the potential to provide a ray of hope for patients afflicted with this malignancy.

#### Colorectal cancer

**Fig. 3:**
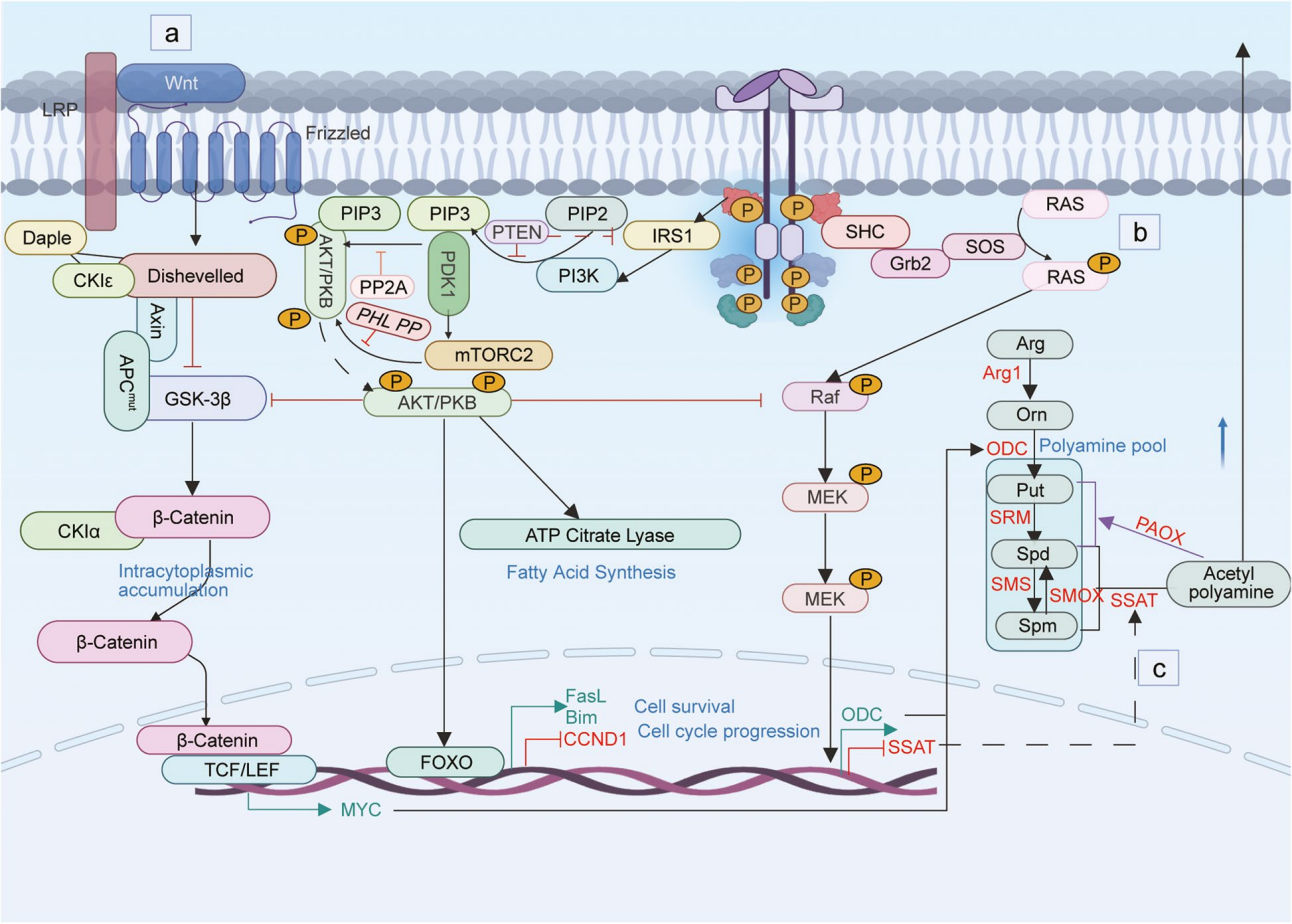
The regulatory pathways of polyamines in CRC. **a** Activation of the Wnt signaling pathway results in the inability of β-catenin to undergo phosphorylation by GSK-3β, ultimately leading to its accumulation within the cell and subsequent nuclear translocation. Upon entering the nucleus, MYC expression is upregulated through binding to TCF/LEF, which will increase the expression of ODC. **B** Excessive stimulation of the RAS signaling pathway results in the stimulation of ODC expression while suppressing the expression of SSAT. **c.** PI3K/AKT pathway can regulat the Wnt and Ras signaling pathways. Elevating the expression of ODC and reducing the expression of SSAT will result in an augmented intracellular pool of polyamines. The elevation in polyamine concentration frequently correlates with an unfavorable prognosis. Created with BioRender.com

In the United States, colorectal cancer (CRC) is the second leading cause of cancer-related deaths and the third most prevalent malignancy worldwide [184]. Notably, approximately a quarter of individuals diagnosed with CRC exhibit synchronous liver metastases, and between 50 and 75% of patients experience liver metastases within a three-year timeframe following initial colon surgery [185]. Despite some advancements in early detection and the use of modern surgical techniques in conjunction with radiotherapy and chemotherapy, the prognosis for CRC patients remains dismal to this day [186]. Therefore, it is crucial to identify the putative molecular mechanisms that drive the progression and metastasis of CRC. The WNT signaling pathway has been identified as an ODC gene regulator [81]. The WNT signaling pathway, when activated, upregulates MYC [82], which acts as a transcriptional activator of ODC [187]. While the WNT cascade is normally suppressed in adult intestinal tissues, it can be disrupted by mutations. In particular, the APC tumor suppressor gene, a key component of the WNT cascade, has been identified as the cause of familial adenomatous polyposis (FAP) [188]. Over 80% of sporadic colorectal cancers are also attributable to APC mutations [189]. These mutations and dysregulated WNT signaling ultimately lead to an increase in MYC activity, ODC expression, and polyamine pools [83]. K-RAS's mutations are deemed to exert a central influence in both the initial stages of malignant progression of colorectal cells and the advanced metastatic ailment [190]. Rial et al., observed that mutant K-RAS upregulates ODC transcription and downregulates SSAT transcription, leading to an increase in polyamine biosynthesis and a decrease in polyamine catabolism (Fig. 3) [83]. Moreover, polyamines can be internalized by cells via the caveolin-mediated endocytosis pathway. The activation of K-RAS results in the phosphorylation of caveolin-1, which effectively enhances the endocytic process and subsequently increases the cellular intake of polyamines [84]. As early as 1986, it has reported that patients exhibiting elevated levels of polyamines in either their blood or urine tend to present with more advanced stages of disease and a poorer prognosis, especially the type of CRC [191, 192]. Elevated levels of polyamines can facilitate the hypusination of eukaryotic initiation factor 5A (eIF5A), thereby triggering the biosynthesis of MYC and initiating a positive feedback loop that consistently promotes the progression of CRC [193, 194]. Disrupting either link within this loop efficiently inhibits CRC development.

#### Pancreatic cancer

Pancreatic cancer (PC) is a malignant neoplasm with an extraordinarily high mortality rate. Pancreatic ductal adenocarcinoma (PDAC) represents the predominant form of PC, comprising over 85% of all diagnosed cases [160]. PDAC is a type of cancer that is primarily driven by inflammation and is characterized by a substantial fibroinflammatory microenvironment [195]. Notably, PDAC's reliance on polyamine confers a number of survival advantages. First, the elevated concentrations of spermidine within the intracellular environment serve as a potential reservoir for the biosynthesis and secretion of substantial amounts of immunosuppressive spermine [196]. Second, it has been observed that spermidine can initiate autophagy by inhibiting EP-300 [85], a process that may promote the survival of PDAC cells in light of their increased metabolic rate, elevated levels of ROS and genomic instability [86, 87]. Elevated levels of spermidine synthesis are linked to increased expression of ODC. The upregulation of ODC is a consequence of the MTAP loss, a frequently observed occurrence in PC [88, 89]. Thirdly, the secretion of polyamines can furnish a plethora of polyamine substrates that can drive desmoplasia through extracellular transglutaminase 2 activity [196]. Desmoplasia is observable in both primary PDAC tumors and their metastatic counterparts [197]. Briefly stating, PDAC proliferation, microenvironment, desmoplasia, and immune function are just a few of the many functions of polyamines that have been elucidated in a large number of published articles. Combinatorial therapies that target polyamine depletion have demonstrated efficacy in inhibiting tumor growth and boosting immune response [198–200]. SBP-101, a Spm analogue that effectively reduces the activity of ODC and slightly promotes the catabolism of polyamine, has currently been evaluated in Phase 2/3 clinical trials for the treatment of PDAC (NCT05254171) [70]. Eventually, this field represents a promising avenue for drug discovery, and the successful development of polyamine-targeted therapies could have far-reaching implications for a variety of human diseases that hinge on polyamines for cellular growth and intercellular communication [201, 202].

#### Prostate cancers

Prostate cancer (PCa) is the most prevalent non-cutaneous neoplasm affecting the male population on a global scale, with an expected incidence of approximately 1.6 million cases and 366,000 deaths per year [203]. Despite advances in therapies targeting the androgen axis, the emergence of castration-resistant prostate cancer (CRPC) among patients is unavoidable [204]. Therefore, there continues to be an urgent need for more effective PCa treatments.

Luminal epithelial cells of the prostate gland exude amounts of acetylated polyamines into the prostatic lumen, rendering them vulnerable to disruptions in associated metabolic pathways [90]. Affronti et. al., have demonstrated that the rapid rate of flux through polyamine biosynthesis in PCa can be utilized in three distinct ways [90]. First, models of both androgen-stimulated and castration-recurrent PCa have revealed that dietary folate depletion is an efficacious means of treatment [205, 206]. Secondly, androgen-sensitive models have shown that the inhibition of the methionine salvage pathway is an effective approach [207]. The current investigation has established that a combination of methionine salvage pathway inhibition and activation of polyamine catabolism is synergistic and highly effective in treating both androgen-sensitive and androgen-independent models of PCa [90]. In addition, a study demonstrates that PGC1-α is a vital regulator in the modulation of PCa cell aggressiveness and that the depletion of PGC1-αinduces a dependence of prostate cancer cells on the polyamine pathway, which promotes metastasis [92]. PGC1-αreduces the expression of MYC and ODC, thereby significantly decreasing intracellular polyamine levels. In other words, PGC1-α effectively suppresses the metastatic properties of prostate cancer cells by downregulating the polyamine biosynthesis pathway. However, Li et al. have reported that the exogenous addition of Spm can impede the proliferation of CRPC [208]. Thus, further investigations are necessary to elucidate the exact function and specific molecular mechanisms underlying the influence of polyamine concentration on the growth and proliferation of prostate cancer cells.

#### Neuroblastoma

Neuroblastoma is a common childhood tumor that originates in the peripheral nervous system. The amplification of the MYCN proto-oncogene has a strong correlation with advanced disease and is the genetic characteristic most consistently associated with ineffective treatment [209, 210]. The oncogene ODC1, which encodes the rate-limiting enzyme in polyamine synthesis and serves as a compelling target [211], is a compelling mediator of MYC effects [187]. Due to the dysregulation of MYC, the activity of ODC is typically upregulated in cancer, resulting in an increase in polyamine levels that promote the rapid proliferation of tumor cells [99]. The administration of DFMO, a selective inhibitor of ODC, has been shown to be an effective strategy for the inhibition of both the occurrence and progression of MYC-associated Neuroblastoma [93–96, 98]. Currently, DFMO has progressed to the Phase 2 stage of clinical trials conducted in neuroblastoma (NCT02395666). Furthermore, it has been demonstrated that probenecid can exert inhibitory effects on the renal clearance of DFMO [212]. Therefore, Schultz et al. also indicated that the addition of probenecid as a supplementary medication during the course of neuroblastoma therapy enhanced the therapeutic outcome of DFMO. In this regard, Michael et al., provided evidence that the expansion of polyamines via the broad deregulation of regulatory enzymes, including ODC1, was a defining characteristic of neuroblastomas exhibiting MYCN amplification, and they identified a strong correlation between elevated levels of ODC1 and unfavorable clinical outcomes in a large patient cohort, including those without MYCN amplification [93]. Furthermore, MYC positively regulates the transcriptional activation of SLC3A2, leading to an increase in cellular uptake of polyamines, in addition to its role in upregulating ODC activity [97]. However, Lodeserto et al. argued that when the level of polyamine in cancer cells surpassed a certain threshold, it triggered the generation of oxidative metabolites which ultimately causes cell death [213]. Thus, they elicited cell death in neuroblastoma cell lines by elevating the polyamine concentration of cancer cells through the utilization of nanospermidine.

## 3. The effect and mechanism of oncogenes and signaling pathway on polyamine

**Fig. 4:**
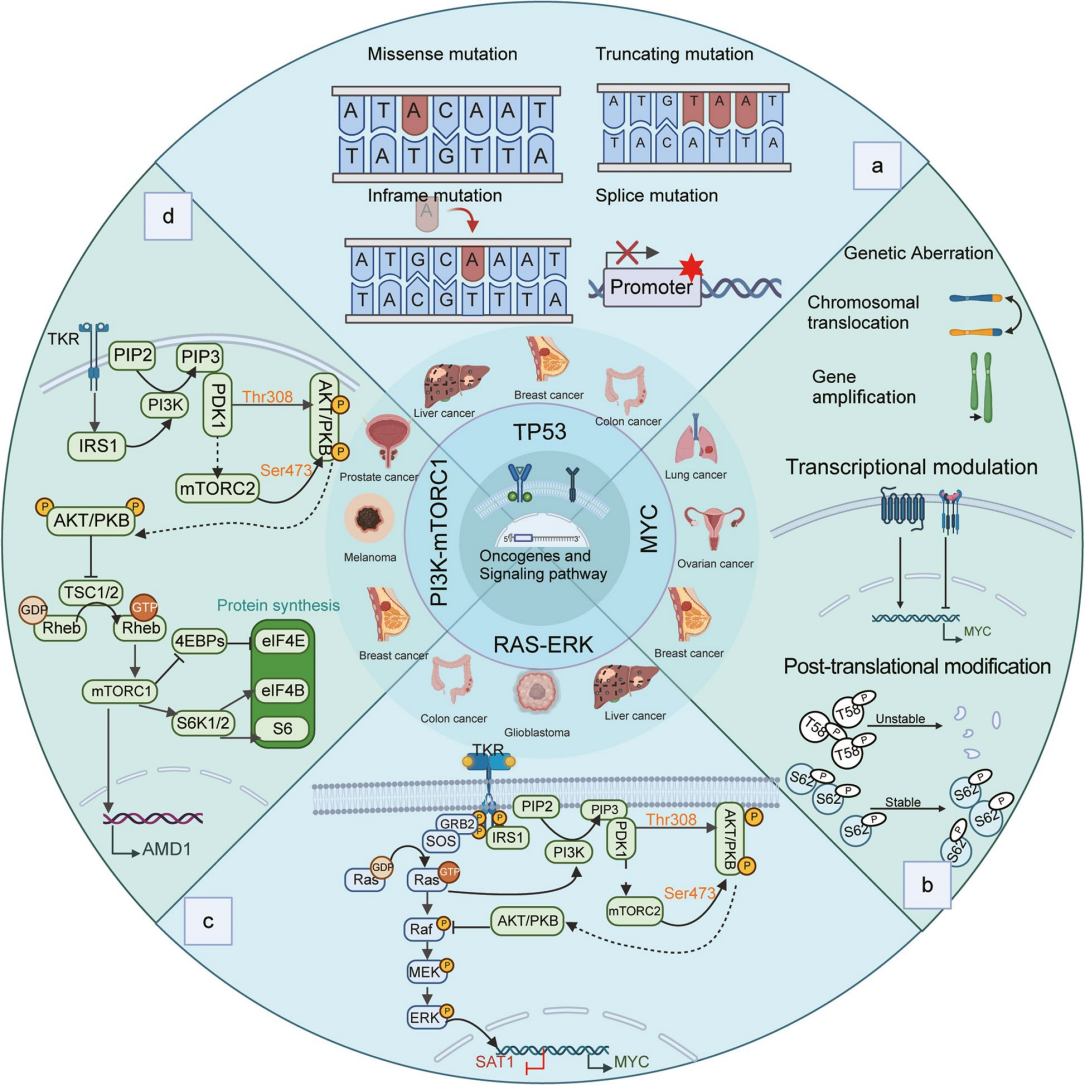
Regulation of polyamine by oncogenes and signaling pathways. **a** The translation product of TP53, known as p53, regulates the expression of ODC, a key enzyme in polyamine synthesis. Mutations in the TP53 gene will have a significant impact on the metabolism and function of polyamines. The tumor suppressor gene TP53 can be inactivated in four ways: Missense mutation; Truncating mutation; Frameshift mutation; Splice mutation. **b** Activated MYC exerts regulatory effects on several crucial enzymes involved in polyamine metabolism, including ODC, SRM and SMS. Genetic aberrations, transcriptional regulation, and protein instability can activate MYC. Increasing phospho-serine 62 (P-S62)-MYC and decreasing phospho-threonine 58 (P-T58)-MYC stabilize MYC protein. **c.** RAS-RAF-MEK-ERK signaling pathway upregulates the expression of ODC and downregulates the expression of SSAT. **d** PI3K-mTORC1 signaling pathway increases the expression of dcAdoMet, thus regulating the biosynthesis of polyamine. Created with BioRender.com

Changes in polyamine concentrations may cause DNA damage [13], which has a close relationship with oncogene dysregulation and the development of cancer. Polyamines are integral components of the downstream target and signaling pathways of numerous oncogenes [75, 165, 214], and the content and activity of polyamines in tumors frequently undergo dramatic alterations [215, 216]. Consequently, a comprehensive examination of the significance of the interaction between polyamines, oncogenes, and signaling pathways in the development and invasion of tumors would yield novel insights into the prevention and treatment of tumors (Fig. 4).

### MYC

The MYC gene cluster, which consists of the c-MYC (MYCC), MYCN, and MYCL genes, has been intensively studied in cancer and developmental biology [217]. MYC activation has been observed in numerous types of cancer, which results from genetic mutations, transcriptional regulation, and protein stabilization.

The first indication of direct interaction between polyamine metabolism and oncogene was the demonstration that the MYC oncogene targeted the transcription of ornithine decarboxylase (ODC) [187]. The stimulation of growth, which results in the elevated expression of MYC, induces an increase in ODC1 mRNA, ODC protein, and ODC activity, thereby supplying the cells with the polyamines required for proliferation [187, 218]. Furthermore, Forshell et al. observed in their research that the stimulation of MYC directly led to the induction of SRM [219]. They also demonstrated that the chemical prevention of B-cell lymphoma via the suppression of SRM represented a promising therapeutic strategy. In addition, the expression of MYC may potentially influence the concentration of Spm through the direct stimulation of SMS [220]. Thus, the MYC signaling pathway is deemed as being among the principal culprits responsible for the dysregulation of polyamine metabolism in cancer [19]. As MYC family members are frequently amplified or overexpressed in cancer, the regulation of polyamine biosynthesis by MYC family genes emerges as a significant contributory factor in various cancer types, such as myeloproliferative neoplasms (MPNs) [221, 222], neuroblastoma [93, 223], CRC [224] and breast cancers [225, 226].

### TP53

The TP53 gene is responsible for encoding the essential tumor suppressor protein p53, which is necessary for the maintenance of normal cellular growth and the prevention of tumorigenesis [227]. The p53 inhibits the expression of key enzymes involved in the urea cycle, resulting in a decrease in the overall rate of urea synthesis and the accumulation of excess ammonia [228]. The excessive accumulation of ammonia can induce a notable decrease in the expression levels of ODC mRNA [229], the rate-limiting factor in polyamine biosynthesis. As a result, the concentration of polyamines within the cell decreases. Moreover, spermine has the ability to induce autophagy by stimulating p53 [230], contributing to the suppression of tumorigenesis. Given the vital role polyamines play in cell proliferation and survival [13, 14], p53 can potentially exert anticancer effects by regulating the urea cycle and suppressing polyamine synthesis [228, 229]. Unfortunately, TP53 mutations are common in the vast majority of human cancers, resulting in impaired anti-tumor activity of the p53 protein and the protein's endowment with oncogenic properties [231, 232].

### PI3K-mTOR complex 1 (mTORC1) signaling pathway

A fundamental signaling molecule that integrates metabolic and growth pathways is the mechanistic target of rapamycin (mTOR) [233, 234], an evolutionarily conserved serine/threonine kinase classified as a member of the phosphoinositide 3-kinase-related kinase (PI3K) family [235]. The central function of mTOR in cellular proliferation has been ascribed to mTORC1 [236], which, upon activation stimulates protein and lipid synthesis while inhibiting autophagy and lysosome formation [235]. In part, these responses are triggered by the phosphorylation of mTORC1 substrates, such as ribosomal S6 kinase 1 (S6K1), eukaryotic translation initiation factor 4E (eIF4E)-binding proteins 1 and 2 (4EBP1/2), and p70S6K1 [236, 237].

Akinyele et al., have confirmed that the modulation of polyamine levels results in significant changes in the phosphorylation levels of 4EBP1 and p70S6K, thereby affecting translation initiation in breast cancer cells, and that the knockdown of the mTOR gene inhibits cell proliferation while simultaneously causing a decrease in putrescine and spermidine content [214]. In addition, it has been shown that the PI3K-mTOR complex 1 (mTORC1) signaling pathway is associated with polyamine metabolism in PCa via the induction of AMD1 expression [91, 238, 239]. Current studies demonstrate that mTORC1 is required for the upregulation of AdoMetDC activity and levels of dcAdoMet, and the suppression of mTOR results in a significant decrease in AdoMetDC activity and intracellular polyamine levels [19, 91]. Initiation of polyamine catabolism also induces a shift in the positioning of mTOR in glioma cells, which has a negative effect on mTOR-related protein synthesis and ultimately leads to apoptosis [240].

### RAS-RAF-MEK-ERK signaling pathway

The RAS/RAF/MEK/ERK signal transduction pathway is essential for cellular communication both within and between cells, regulating fundamental cellular processes such as growth, survival, differentiation, and oncogenesis [241]. As a substrate of RAS, ODC is composed of four distinct splice variants, each of which contains unique intronic sequences. Notably, all splice variants can be translated by means of IRES (internal ribosome entry site) mechanisms [242]. When the RAS signaling pathway is suppressed in non-cancerous cells, the IRES activity of ornithine decarboxylase (ODC) is reduced; however, in cells that have undergone RAS transformation, there is a noticeable rise in the activity of ODC IRES, leading to heightened levels of ODC activity [243]. Notably, RAS is highly prone to mutations in various types of human cancers, resulting in ODC activity [242]. Activated KRAS substantially enhances the uptake of polyamines, thereby altering the localization of the urokinase-type plasminogen activator receptor (uPAR) ligand and resulting in the stimulation of SRC [243]. When stimulated, the SRC enzyme catalyzes the addition of phosphate groups onto caveolin-1, an inhibitor of caveolar endocytosis [84], which has been shown to be associated with increased polyamine uptake in CRC cells [84]. Moreover, the impact of KRAS on the inhibition of SSAT has been proven through its disruption of the peroxisome proliferator-activated receptor-γ (PPARγ) mediated transactivation of SAT1. This interference enables transformed cells to maintain enhanced polyamine levels [19, 244].

## Summary

Our work focuses primarily on summarizing the function and mechanism of polyamines in maintaining health and contributing to disease. A brief summary of the origin and metabolism of polyamine is also provided. Ultimately, the manuscript describes the influence of oncogenes and signaling pathways on polyamine metabolism in a variety of cancer types via enzyme modulation. We hope that this review will provide some insights for the further elucidation and empirical study of polyamines.

## Data Availability

No original datasets were generated for this review. All data supporting the information given here can be found in the references cited within the paper.
